# Anatomical differences in nociceptor neurons sensitivity

**DOI:** 10.1186/s42234-022-00088-w

**Published:** 2022-04-06

**Authors:** Theo Crosson, Sebastien Talbot

**Affiliations:** Département de Pharmacologie et Physiologie, Faculté de Médecine, Université de Montréal, Montréal, QC Canada

**Keywords:** Nociceptor neurons, Neuro-immunity, Dorsal root ganglia, Jugular Nodose ganglia, Brain-Derived Neurotrophic Factor, Nerve growth factor, Ntkr1, TRPV1, TRPM8, TRPA1

## Abstract

**Background:**

Dorsal Root Ganglia (DRG) neurons are derived from the neural crest and mainly innervate the skin, while Jugular Nodose Complex (JNC) neurons originate from the placode and innervate internal organs. These ganglia are composed of highly heterogeneous groups of neurons aimed at assessing and preserving homeostasis. Among other subtypes, nociceptor neurons are specialized in sensing and responding to environmental dangers. As form typically follows function, we hypothesized that JNC and DRG neurons would be phenotypically and transcriptomically different.

**Methods:**

Mouse JNC and DRG neurons were cultured *ex vivo.* Using calcium imaging, qPCR and neurite outgrowth assay, we compared the sensitivity of JNC and DRG neurons. Using *in-silico* analysis of existing RNA sequencing datasets, we confronted our results to transcriptomic differences found between both ganglia.

**Results:**

We found drastically different expression levels of Transient Receptor Potential (TRP) channels, growth factor receptors and neuropeptides in JNC and DRG neurons. Functionally, naïve JNC neurons’ TRP channels are more sensitive to thermal cues than the ones from DRG neurons. However, DRG neurons showed increased TRP channel responsiveness, neuropeptide release and neurite outgrowth when exposed to Nerve Growth Factor (NGF). In contrast, JNC neurons preferentially responded to Brain-derived neurotrophic factor (BDNF).

**Conclusion:**

Our data show that JNC and DRG neurons are transcriptomically and functionally unique and that pain sensitivity is different across anatomical sites. Drugs targeting NGF signaling may have limited efficacy to treat visceral pain. Bioelectronics nerve stimulation should also be adjusted to the ganglia being targeted and their different expression profile.

**Supplementary Information:**

The online version contains supplementary material available at 10.1186/s42234-022-00088-w.

## Background

Nociceptor neurons, a subtype of sensory neurons, form a key line of defense against environmental dangers. They detect a broad range of thermal, mechanical, and chemical threats and respond by means of protective reflexes and by modulating the cells of the microenvironment. Nociceptor neurons can be differentiated based on their expression profiles (Prescott et al., 2020; Usoskin et al., 2015; Kupari et al., 2019; Kaelberer et al., 2020; Sapio et al., 2020), degree of myelination, type of cues to which they are sensitive (Crosson et al., 2019), the reflexes they initiate, the anatomical location of their soma, or the anatomical site they innervate (Mazzone & Undem, 2016). For instance, dorsal root ganglia (DRG) neurons are derived from the neural crest and innervate the skin, muscles, and joints, while the Jugular Nodose Complex (JNC) neurons originate from the neural crest (Jugular) and placode (Nodose) and innervate internal organs (Chavan et al., 2017; Baker & Schlosser, 2005).

Nerve growth factor (NGF) was discovered for its role in the development of the peripheral nervous system (Levi-Montalcini & Hamburger, 1951; Crowley et al., 1994), including nociceptors. In adulthood, NGF is released by glial, epithelial, or immune cells (Lindholm et al., 1987; Heumann et al., 1987; Leon et al., 1994; Donnerer et al., 1992; Woolf et al., 1994; Longo et al., 2013; Braun et al., 1999; Stanzel et al., 2008) in response to injury or inflammation. In turn, by acting on its specific receptors, TrkA, which is highly expressed on nociceptors, NGF promotes nerve regeneration (Onger et al., 2017; Sofroniew et al., 2001), tissue innervation (Kinkelin et al., 2000; Ghilardi et al., 2012; Reinert et al., 1998), and increases nociceptors sensitivity (Ji et al., 2002; Chuang et al., 2001; Shu & Mendell, 1999; Nicholas et al., 1999; Koltzenburg et al., 1999; Zhang et al., 2005). As a result, several drugs, notably monoclonal antibodies, targeting NGF signaling are developed to treat chronic pain (Wise et al., 2021). Despite TrkA expression in JNC neurons (Verge et al., 1992; Zhuo & Helke, 1996), the impact of NGF on vagal nociceptors remains controversial. On the one hand, NGF does not affect the survival of JNC neurons (Lindsay & Rohrer, 1985; MacLean et al., 1988), but it was found to increase their expression of Substance P (SP) (MacLean et al., 1988; Hunter et al., 2000).

As the site they innervate (skin, joints) can be readily studied using standard behavioral tests (i.e., Von Frey, Hot plate), DRG neurons’ function, sensitivity and biophysical properties is well characterized. In contrast, airway or gut pain is difficult to assess; leaving JNC neuron’s function poorly defined. To address these shortcomings, specific ganglia can be isolated, cultured *ex vivo* and neurons’ sensitivity to various stimuli can be studied using calcium microscopy as a mean to infer pain sensitivity. Using such an approach, we discovered that JNC neurons have an increased sensitivity to thermal stimuli. We also found that while NGF enhanced DRG neurons sensitivity and neurite outgrowth of nociceptors, it had limited effect on JNC neurons.

Our data shows that JNC and DRG neurons are transcriptomically and phenotypically different, revealing that targeted pain blocking molecules, such as NGF blockers, will likely be ineffective to blunt visceral pain. In addition, JNC neurons express different neuropeptides than the ones from DRG. These data suggest that JNC neuron immunomodulatory actions are also likely to be different from the one produced by DRG neurons. Altogether, these findings support an anatomical-dependent neuronal control of immunity and pain.

## Results and discussion

Upon sensing danger, nociceptors initiate defensive reflexes (ranging from withdrawal response to coughing); and release neuropeptides, which regulate vasodilation, mucus secretion, and local infiltration of immune cells (Chiu et al., 2012). The resulting outcome of such neuro-immune interplay is still ill-defined, but data support neuropeptides as blockers of T_H_1-mediated immunity but also as drivers of T_H_2 immune responses (Talbot et al., 2015; Crosson et al., 2021; Foster et al., 2017; Trankner et al., 2014; Chu et al., 2020; Baral et al., 2018; Chiu et al., 2013; Serhan et al., 2019).

**Fig. 1:**
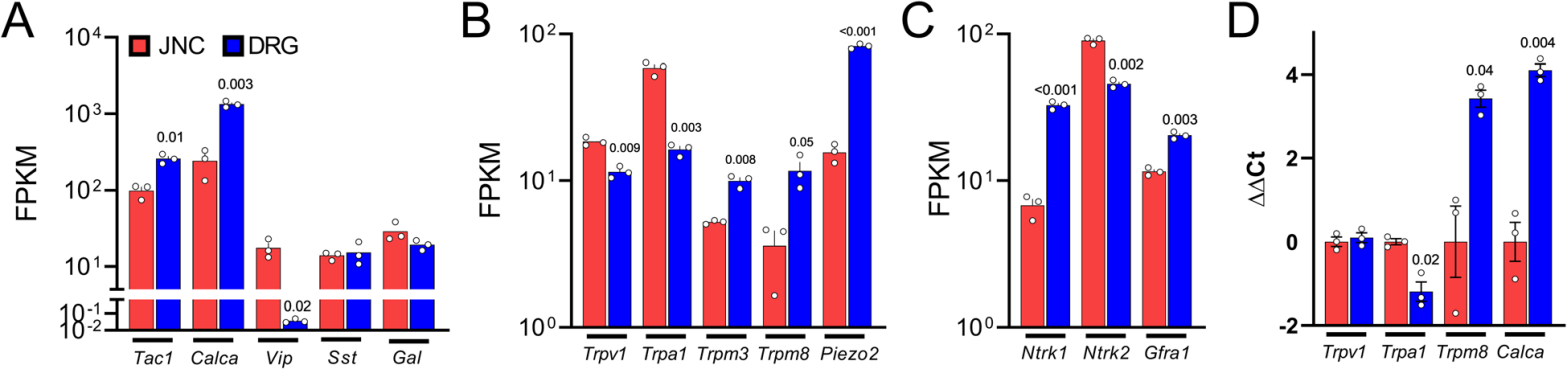
Lumbar and vagal neurons transcriptomes are different. (**A**-**C**) The whole jugular nodose ganglia complex (JNC; n=3; red) and dorsal root ganglia (DRG; n=3; blue) mouse transcriptomes were profiled using RNA sequencing (RNA-seq; Illumina HiSeq 2500) by *Kaelberer et al*., 2020. *In-silico* analysis of the neuron's transcriptome revealed that naïve mouse JNC nociceptor neurons express different neuropeptides (**A**), ion channel receptors (**B**), and growth factor receptors (**C**) than the one found in DRG. JNC neurons are enriched for *Vip*, *Trpv1*, *Trpa1*, and *Ntrk2*, while DRG neurons show higher higher levels of *Calca*, *Tac1*, *Trpm8*, *Trpm3*, *Piezo2*, *Gfra1* and *Ntrk1*. (**D**) Gene expression was also assessed by RT-qPCR after 24 hours of culture (**D**). ΔΔCt was calculated in comparison to JNC. *Trpm8* and *Calca* were enriched in DRG neurons, while *Trpa1* was enriched in JNC neurons (**D**). *Expression across datasets is shown as Fragments Per Kilobase of transcript per Million reads mapped* *(FPKM)* *(****A***-***C****)* *or* *ΔΔCt* with JNC as the control condition *(****D****)*.* Data are shown as means* ± *S*.*E*.*M*. *N=3/groups*. *P*-*values* *are shown in the figure and determined by unpaired multiple Student* *t*-test *with Hidom-Sidak corrections* *(****A***-***D****)*. *Experimental details were defined in Kaelberer et al.,* 2020 (***A***-***C***)

As lumbar and vagal neurons innervate different mucosa, we hypothesized that these neurons are tuned to detect different modalities. As a result, we expect these neurons to express different ion channel receptors and neuropeptides. To uncover whether it is the case, we proceed to the *in-silico* analysis of the RNA sequencing dataset published by Kaelberer and colleagues (Kaelberer et al., 2020). As posited, naïve mouse JNC neurons express different neuropeptides (Fig. 1A), ion channel receptors (Fig. 1B), and growth factor receptors (Fig. 1C) than the ones found in DRG. For instance, JNC neurons are enriched for *Vip,* and *Ntrk2,* while DRG neurons show higher levels of *Calca,* and *Ntrk1 *(Fig. 1A and C). Furthermore, naïve JNC neurons overexpress the heat receptors *Trpv1* and cold/heat receptor *Trpa1*, while the DRG neurons are enriched for the cold receptor *Trpm8* and heat receptor *Trpm3* (Fig. 1B). Of note, these genes were selected as they are prototypical markers of sensory neurons’ functionality.

Kaelberer and colleagues sequencing used freshly harvested ganglia. Since JNC or DRG neuron phenotyping is often carried out with primary cultures, we’ve investigated if cultured neurons show the same expression pattern as freshly dissociated cells. After 24h in culture, JNC neurons show higher *Trpa1* expression than DRG neurons and lower one for *Trpm8* and *Calca* (Fig. 1D). *Trpv1* expression was comparable in both cultures. Overall, these results largely match the one from the sequencing of fresh ganglia - but differs slightly for *Trpv1*.

**Fig. 2:**
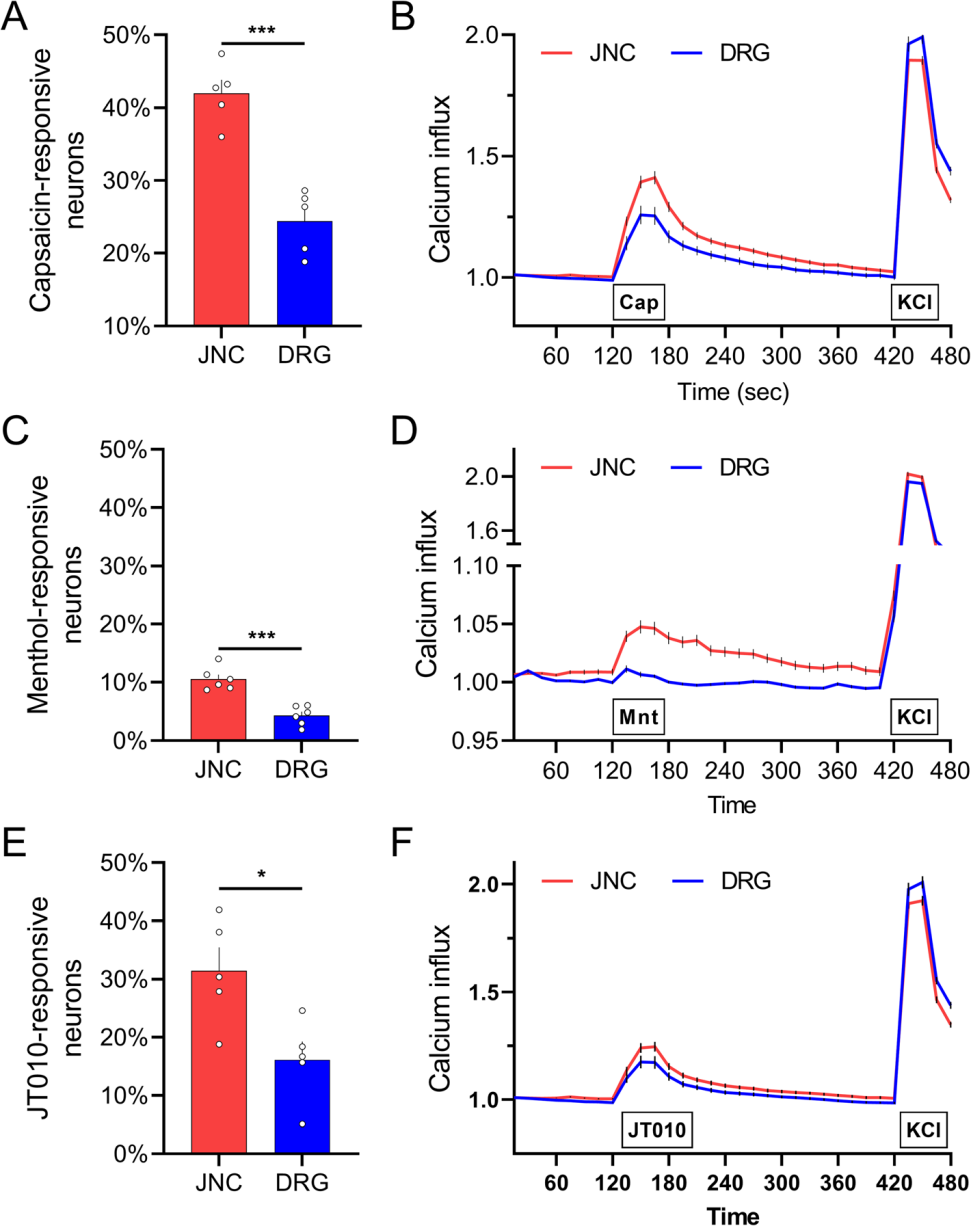
JNC neurons are more sensitive to TRPV1, TRPA1 and TRPM8 stimulation. Naïve C57BL/6 mouse JNC and DRG neurons were harvested, cultured (2,500 neurons/plate) for 24h, loaded with a calcium responsive dye (Fura-2AM), and sequentially exposed to the TRPV1 agonist capsaicin (300nM; **A-B**), the TRPA1 agonist menthol (100uM; **C-D**), or the TRPA1 agonist JT010 (1uM, **E-F**) followed by KCl (40mM; positive control; **A-F**). The number of responsive (**A, C, E**) cells to capsaicin, menthol, and JT010 is increased in JNC neurons. The average amplitude of responses are shown in (**B, D, F**). *Data are shown as mean ± S.E.M. Number of dishes tested is shown. N are as follows:* ***A-C-E****: n=5-6 dishes/group*, ***B-D-F****: n=290-1200 neurons/group. P-values are shown in the figure and determined by unpaired Student's t-test (****A, C, E****)*

To assess whether these transcriptomic differences translate to physiology, we set up a side-by-side culture system to compare the sensitivity of JNC and DRG neurons (2,500 neurons/dish) to menthol (TRPM8 agonist, 100 μM), capsaicin (TRPV1 agonist, 300 nM) and JT010 (TRPA1 agonist (Takaya et al., 2015), 1μM). Using calcium microscopy as a proxy for neuron’s sensitivity, we found that in comparison to DRG, JNC neurons cultures show higher numbers of calcium responsive neurons when exposed to capsaicin (Fig. 2A-B), menthol (Fig. 2C-D) and JT010 (Fig. 2E-F).

While the respective responses to capsaicin and JT010 matches with the higher expression of *Trpv1* and *Trpa1* in JNC neurons, the menthol data are opposite to one predicted by the transcriptomic profile of *Trpm8* (Fig. 1B). Such discrepancy between *Trpm8* gene expression and calcium response to menthol demonstrates that bulk gene expression cannot be used as a sole indicator of neuronal sensitivity. Indeed, the activation of sensory neurons also depends on the number of neurons expressing the thermoreceptor, the relative membrane expression of each ion channel on a given neuron, their phosphorylation status, their activation threshold and the one of the voltage-gated sodium and calcium channels bore by these neurons. In addition, reports suggest the possible action of menthol on TRPA1 (Karashima et al., 2007; Lemon et al., 2019; Macpherson et al., 2006), which could explain the inconsistency we observed between gene *Trpm8* expression and menthol responsiveness.

**Fig. 3:**
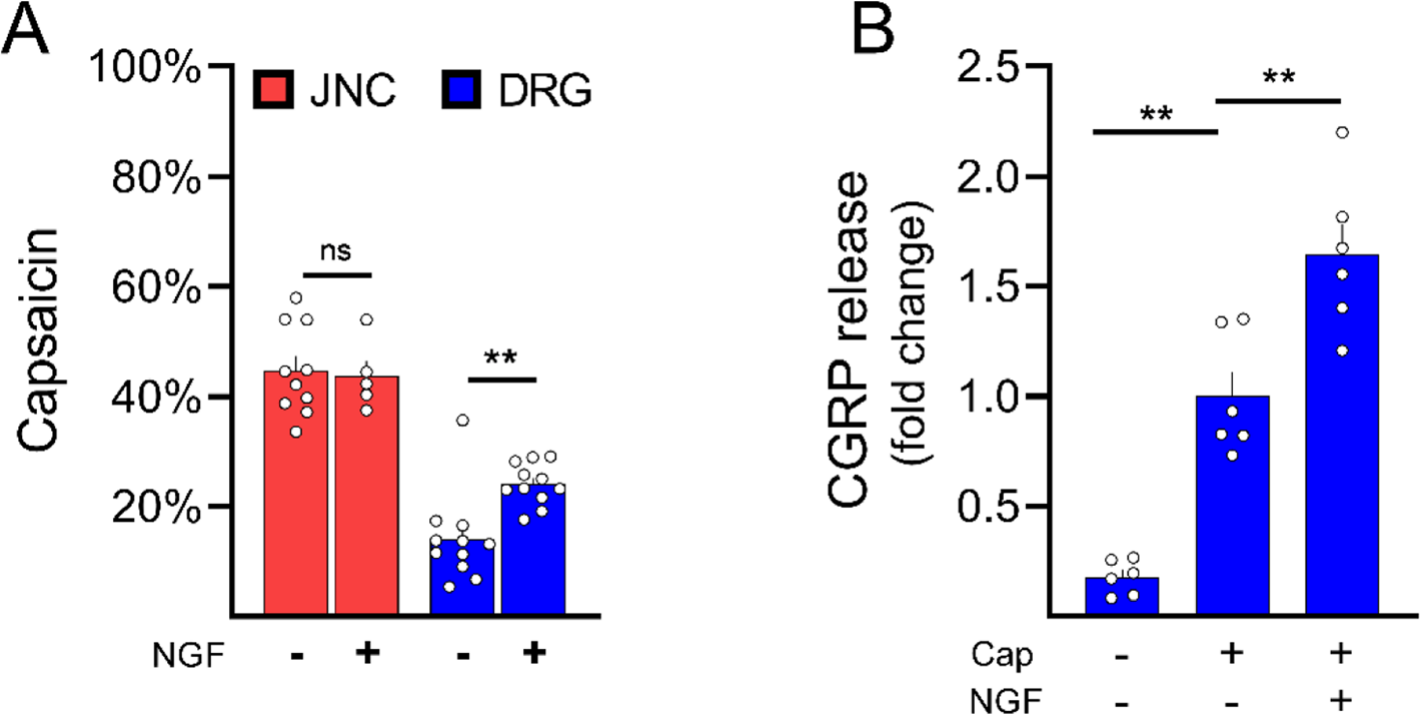
JNC neurons are insensitive to NGF. Naïve C57BL/6 mouse JNC and DRG neurons were harvested, cultured for 24h in the presence of Nerve Growth Factor (NGF, 50 ng/mL). The cells were then loaded with a calcium responsive dye (Fura-2AM) and sequentially exposed to capsaicin (100 nM) followed by KCl (40mM; positive control; **A)**. The addition of NGF increased the number of capsaicin-responsive neurons in DRG cultures but not in the one from the JNC (**A**). Similarly, NGF increased capsaicin-induced CGRP release from cultured DRG neurons **(B)**. *Data are shown as mean ± S.E.M. Number of dishes tested is shown. N are as follows:* ***A****: n=5-10 dishes/group from two independent experiments*, ***B****: n=6 wells from different animals/group from two independent experiments. P-values are shown in the figure and determined by unpaired Student's t-test (****A****) or one-way ANOVA post hoc Dunnett (****B****)*

We next sought to investigate the sensitivity of nociceptors to neurotrophins, including Nerve Growth Factor (NGF) that is widely used in nociceptor cultures. By acting on its receptor TrkA (*Ntkr1)*, NGF is known to promote kinase activation (Ji et al., 2002; Zhang et al., 2005), ion channel phosphorylation, and increased trafficking to the plasma membrane (Ji et al., 2002; Stein et al., 2006), all of which resulting in heightened TRP channels sensitivity. First, we noticed that *Ntkr1* is preferentially expressed in DRG neurons (Fig. 1C) and, as expected, adding NGF to the DRG cultures enhanced their responsiveness to capsaicin (Fig. 3A). In contrast, NGF surprisingly had no impact on JNC neurons’ responses to capsaicin.

Another feature of neuron activation is their release of neuropeptides. This allows sensory neurons to modulate the synaptic transmission of electrical signals and play a crucial part in promoting pain chronicity. It is also an essential feature by which nociceptor neurons modulate immune cells activity. Similar to calcium flux, we discovered that NGF supplementation increased capsaicin-induced CGRP release from DRG neurons (Fig. 3B). Interestingly, the increased capsaicin sensitivity and CGRP release in response to NGF was not associated with increase in *Trpv1* and *Calca* transcripts (Supplementary Figure 1A-B). This result is in line with previous findings suggesting that nociceptor sensitization by NGF exclusively relies on post-transcriptional mechanisms (Ji et al., 2002).

**Fig. 4:**
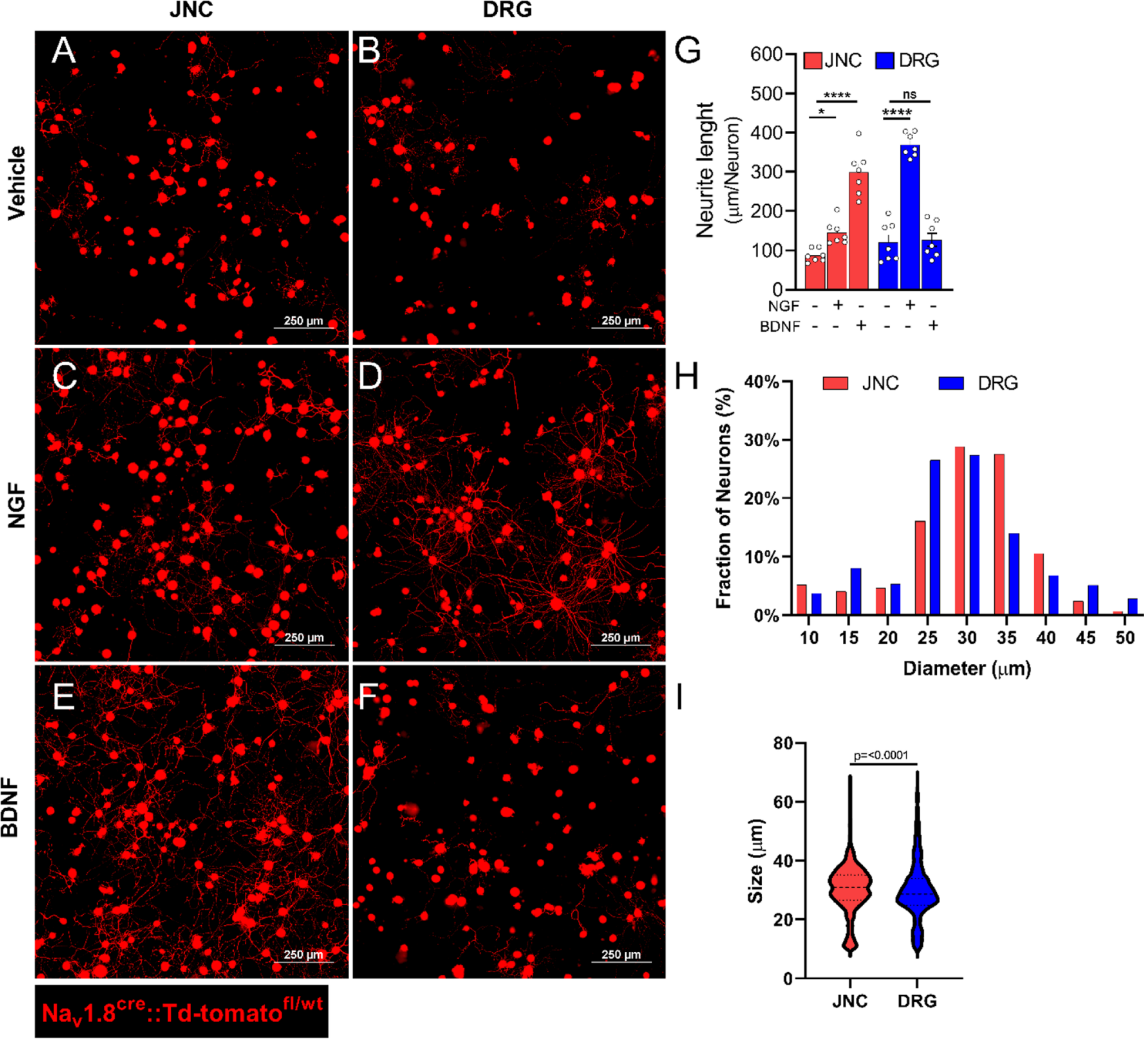
Lumbar and vagal neurons are phenotypically different and respond differently to NGF and BDNF. Na_V_1.8^cre^::Td-tomato^fl/wt^ (red) mice JNC (**A, C, E**) and DRG (**B, D, F**) nociceptor neurons were harvested, and cultured (1,500 neurons/plate) for 24h in the presence of vehicle (**A**-**B**), Nerve Growth Factor (NGF, 50 ng/mL; **C**-**D**), or Brain-Derived Neurotrophic Factor (BDNF, 50 ng/mL; **E**-**F**). In comparison to vehicle, NGF increased neurite outgrowth in cultured DRG neurons, and had limited impact in JNC neurons (**G**). BDNF increased neurite outgrowth in cultured JNC neurons but had no impact in DRG neurons (**G**). In vehicle-exposed culture, JNC neurons have a larger diameter than DRG neurons (**H**-**I**; n>1000 neurons). *Data are shown as mean ± S.E.M. N=7 dishes/group from two independent experiments* (***A***-***I***). *P-values are shown in the figure and determined by one-way ANOVA post hoc Dunnett* (***G***) *or Mann-Whitney Test* (***I***). *Scale = 250 μm* (***A***-***F***). NaV1.8^+^ *nociceptors are labeled in red* (***A***-***F***)

So far, we probed NGF-mediated sensitization by measuring whether the neurons show heightened responsiveness to capsaicin (calcium influx, neuropeptide release). Along with such an important feature of pain-sensing and transmission, we next sought to test whether NGF modulates JNC and DRG neurite outgrowth. The latter is a process characterized by the growing or branching of neurons in response to guidance cues, which occurs in the developing nervous system and during nerve regeneration. As previously reported, NGF increased the neurite length of cultured DRG Na_V_1.8 positive nociceptors (Fig. 4B, D, G). In line with the other features we had previously tested, we found that neurite outgrowth of JNC nociceptors were very mildly affected by NGF (Fig. 4A, C, G), but was strongly enhanced by Brain-Derived Neurotrophic Factor (BDNF; Fig. 4E, G). DRG nociceptors’ neurite growth was not changed by BDNF supplementation (Fig. 4F, G). While NGF is an important driver of neurogenic inflammation (Donnerer et al., 1992; Woolf et al., 1994; Longo et al., 2013; Braun et al., 1999), our data suggest that it would have limited impact on vagal sensory neuron activity and growth, but would rather target DRG and sympathetic neurons (Kinkelin et al., 2000; Ghilardi et al., 2012; Aloe et al., 1992).

**Fig. 5:**
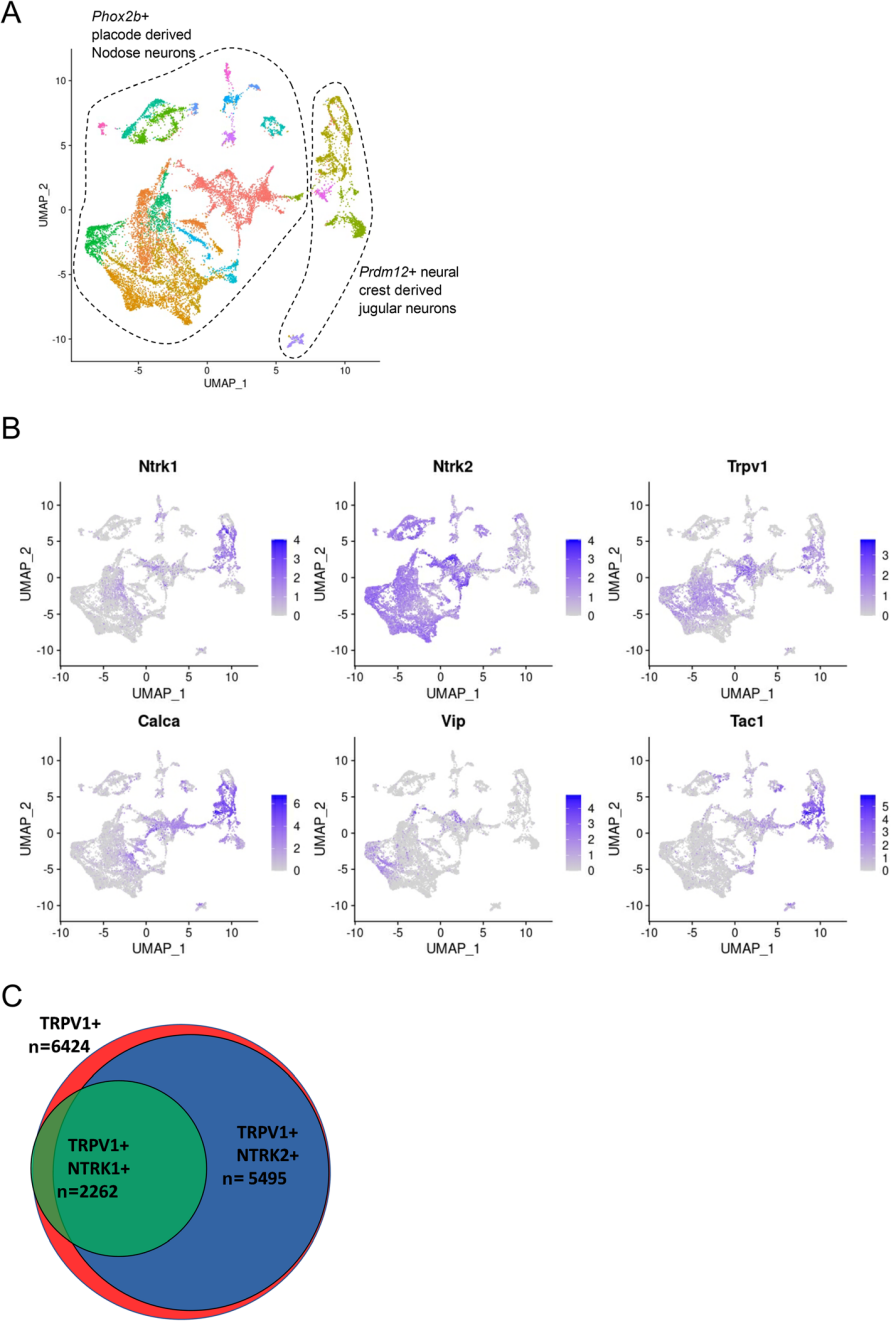
Nodose neurons have low expression of *Ntrk1* but are enriched for *Ntrk2*. *In-silico* analysis of single-cell RNA sequencing of 15,142 *Slc17a6*^*+*^* (Vglut2)* JNC sensory neurons (**A**). *Phox2b*^+^ neurons are derived from the placode which are designated as nodose neurons, while *Prdm12*^+^ neurons are derived from the neural crest and indicate jugular neurons (**A**). Uniform Manifold Approximation and Projection (UMAP) were generated, featuring normalized expression of neurotrophins receptors (*Ntrk1, Ntrk2*), ion channel receptor (*Trpv1*) and neuropeptides (*Calca, Vip, Tac1*; **B**). *Calca, Tac1, Ntrk1* are enriched in jugular (neural crest) neurons whereas *Vip* and *Ntrk2* are preferentially expressed by nodose (placode) neurons **(B)**. *Trpv1* (*n*=6424) is expressed in jugular and nodose neurons, *Ntkr2*^*+*^*Trpv1*^*+*^ neurons (*n*=5495) are 2.4-fold more abundant than *Ntkr1*^*+*^*Trpv1*^*+*^ (*n*=2262) neurons (**C**). Experimental details were defined in (Prescott et al., 2020)

Since NGF showed a limited, but not null, effect on JNC nociceptor neurite growth, we hypothesized that only a subtype of JNC neurons would be sensitive to this neurotrophin (Nassenstein et al., 2010). To address whether this is the case, we analyzed Prescott *and colleagues* (Prescott et al., 2020) single JNC neurons RNA sequencing dataset. We found that *Calca*, *Tac1*, *Ntrk1* are enriched in jugular (neural crest derived) neurons, considered transcriptomically close to DRG neurons (Prescott et al., 2020; Kupari et al., 2019), whereas *Vip* and *Ntrk2* are preferentially expressed by nodose (placode derived) neurons (Fig. 5 A-B). Within the JNC, *Trpv1* (*n*=6424) is expressed in jugular and nodose neurons (Fig. 5C). However, *Ntkr2*^*+*^*Trpv1*^*+*^ neurons (*n*=5495) are 2.4-fold more abundant than *Ntkr1*^*+*^*Trpv1*^*+*^ (*n*=2262) neurons (Fig. 5C). Along with our calcium imaging recordings, these data suggest that the JNC contains a large population of Vip^+^ capsaicin-responsive nodose neurons that are insensitive to NGF. In line with the functional and gene expression data we described here, this is an important finding as JNC neuron’s function is typically studied using *ex vivo* cultures supplemented with NGF. Thus, JNC cultures would likely be best model using other neurotrophins. JNC neurons (Fig. 1C) and specifically nodose neurons (Fig. 5C) overexpress the BDNF receptor *Ntkr2,* and BDNF enhance their neurite growth (Fig. 4E). Given that BDNF is also released in inflammatory contexts (Braun et al., 1999), it is likely to impact vagal nociceptor phenotype to a larger extent than NGF.

Finally, we found that cultured Na_V_1.8 positive JNC nociceptors are larger than DRG nociceptors (Fig. 4H-I). These data further support a different composition of the neuronal subtypes between these two ganglia, and this is likely a function of the mucosal danger each is exposed to. Thus, along with the anatomical differences in nociceptor neurons’ ability to sense danger and the molecule driving their sensitization, we also found a substantial discrepancy in the neuropeptide produced by JNC and DRG neurons. Thus, DRG neurons preferentially express SP and CGRP while JNC neurons, specifically the one from the nodose, express VIP (Figs. 1A and 5). Interestingly, these three neuropeptides are reported to regulate immune responses, but their role differs depending on the type of inflammation (T_h_1, T_h_2) and the type of immune cells involved (Foster et al., 2017).

## Conclusions

By innervating locations ranging from skin to visceral mucosa, sensory neurons are exposed to different environments and types of danger. Here, we found significant transcriptomic and phenotypic differences between JNC and DRG neurons. In addition to these, our data revealed that JNC neurons have an intrinsic and heightened capacity to sense heat and cold dangers.

System modelling using *ex vivo* culture systems of JNC or DRG should take into account that JNC and DRG neurons differentially respond to neurotrophins, DRG being more sensitive to NGF, while JNC is more sensitive to BDNF. The management of visceral and skin inflammation and pain should also be tailored differently. Among other differentiator, the selective targeting of these growth factor receptors could help preferentially act on one of these systems while sparing the other. Finally, bioelectronics nerve stimulation should also be adjusted to ganglia being targeted and, depending on the pathological situation, could be accompanied by pharmacological blockers of the NGF or the BNDF receptors.

## Methods

### In silico analysis of whole DRG and JNC transcriptome

RNA sequencing data of mouse nociceptors expressed as Fragments per Kilobase Per Million (FPKM) were downloaded from NCBI Gene Expression Omnibus (GSE141395). Experimental procedures are detailed in the original study published by Kaelberer et al (Kaelberer et al., 2020). The authors extracted whole JNC and DRG from C57BL6 mice, which were then flash-frozen in liquid nitrogen before RNA isolation and sequenced on an Illumina HiSeq 2500. TopHat was used to map reads to mouse reference genome mm9. Genes coding for neuropeptides, ion channels and neurotrophins receptors were processed using Microsoft Excel.

### In-silico analysis of JNC neurons single-cell transcriptome

Prescott et al. (2020) generated single cell sequencing data of nodose ganglia cells from 40 mice using 10X Genomics platform. The data was downloaded from NCBI Gene Expression Omnibus (GSE145216) and analyzed using Seurat. Neuronal cells were selected based on *Slc17a6* (Vglut2) expression (raw count ≥ 2). Standard workflow was used for quality control, preprocessing, normalization and clustering (resolution = 0.5, PCs = 1:30). Neurons were considered positive for other markers if their raw count was ≥ 1.

### Animals

Mice were housed in standard environmental conditions (12h light/dark cycle; 23oC; food and water ad libitum) at facilities accredited by CCPA. Parental strain C57BL6 (Jax, # 000664) and Td-Tomato^fl/fl^ (Jax, # 007908) were purchased from Jackson Laboratory. Parental strain Nav1.8^cre/cre^ mice were generously supplied by Professor John Wood (UCL). Male and female mice C57Bl6 and Nav1.8^cre/wt^::Td-Tomato^fl/wt^ were bred in-house and used between 6 and 12 weeks of age.

### Neuron culture

Mice were sacrificed and JNC ganglia and DRG were dissected out into ice-cold DMEM medium (Corning, # 10-013-CV), completed with 100 U/mL penicillin and 100 μg/mL streptomycin (Fisher, # MT-3001-Cl), and 10% FBS (VWR, #10799-390). JNC ganglia were pooled from several mice. Cells were then transferred in PBS completed with 1 mg/mL collagenase IV (Sigma, #C5138) + 2.4 U/mL dispase II (Sigma, # 04942078001) and incubated for 80 minutes at 37°C. Ganglia were triturated with glass Pasteur pipettes of decreasing size in supplemented DMEM medium, then centrifuged (200g) over a 15% BSA gradient in PBS to eliminate debris. Neurons were then plated on Laminin (Sigma, # L2020) coated cell culture dishes. The cells were cultured at 37° with Neurobasal-A medium (Gibco, # 21103-049) 0.01 mM AraC (Sigma, # C6645) and 200nM L-Glutamine (VWR, # 02-0131), without neurotrophins unless otherwise indicated. Culture densities and durations are further described below for each application.

### Calcium microscopy

2,500 neurons from C57BL6 DRG or JNC in a 10ul drop were plated on laminin-coated glass bottom 35mm dishes (ibidi #81218). 1ml of Neurobasal media supplemented or not with 50ng/mL Nerve Growth Factor (NGF, ThermoFisher #13257019) was added after 1-hour incubation at 37°. Neurons were then cultured overnight before being used for calcium imaging. Cells were loaded with 5μM Fura-2-AM (Biovision #125280) at 37°C for 45-60min in the culture medium then washed into Standard Extracellular Solution (SES, 145 mM NaCl, 5 mM KCl, 2 mM CaCl2, 1 mM MgCl2, 10 mM glucose, 10 mM HEPES, pH 7.5), and imaged at room temperature. Capsaicin 100nM or 300nM (Tocris #0462), Menthol 100uM (medchemexpress #HY-N1369), JT010 1uM (Sigma #SML1672) were prepared in SES and were flowed (30sec) directly onto neurons using perfusion barrels followed by buffer washout (5 minutes). 40mM KCl solution was then flowed on the cells for 20 seconds. Cells were illuminated by a UV light source (Cool LED, pE-340) alternating 340 nm and 380 nm excitation, and a camera (Photometrics Prime 95B 25mm) captured fluorescence emission (515/45nm) with a 20X objective. For analysis, circular Regions of interest (ROI) were manually drawn on neurons based on their 380nm fluorescence. 340/380 fluorescence ratios were then calculated and exported. Microsoft Excel was used for further analyses (Microsoft, USA). Basically, 380/340 ratio values for each ROI were normalized by their baseline. Neurons were considered responsive if the fluorescence ratio increased by at least 10% within 1 minute after injection. Live neurons were defined based on their response to KCl.

### CGRP release

Ten thousand neurons from C57BL6 mice DRG were plated on laminin-coated 96 well plates in 200ul media supplemented or not with 50ng/mL NGF. Biological replicates were made using a different mouse’s preparation for each replicate. After 48 hours of culture, neurons were washed one time with a fresh Neurobasal medium. The medium was then replaced by Neurobasal media, either with or without 300nM capsaicin, before incubation at 37° for 20 minutes. Culture media were then harvested, centrifuged at 700g to remove cellular debris, and used freshly for CGRP Elisa (Cayman #589001) following manufacturer instructions.

### Neurite growth

One thousand five hundred neurons from Nav1.8^cre/wt^::Td-Tomato^fl/wt^ mice DRG or JNC in a 15 ul drop were plated on laminin-coated glass bottom 35mm dishes. 1ml of Neurobasal media supplemented or not with 50ng/mL Nerve Growth Factor (NGF) or Brain Derived Neurotrophic factor (BDNF, Peprotech #450-02) was added after 1-hour incubation at 37°. Pictures of the whole plating area were taken after 24 hours of culture using a 20X objective to collect Td-Tomato fluorescence (Excitation 554/23nm; Emission:609/54nm). For the analysis of neurite outgrowth, an in-house developed method was used. Using Nikon Elements software, a fluorescence threshold was used to define the Td-tomato positive neurites and soma. The somas were then defined and excluded based on fluorescence intensity, size, and circularity. The total size of neurites was then divided by the number of somas for each culture dish. The soma diameters of the culture without neurotrohpin  were used to compare JNC and DRG nociceptors size distribution.

### RT-qPCR

Five thousand neurons from C57BL6 mice DRG or JNC were plated on laminin-coated 96 well plates in 200ul media supplemented or not with 50ng/mL NGF. Biological replicates were made using a different mouse’s preparation for each replicate. After 24 hours of culture, the culture medium was removed, and the cells harvested in 500ul Trizol (Thermofisher #15596018). RNA was then extracted using the kit PureLink™ RNA Micro Scale (Thermofisher # 12183016) following manufacturer’s instructions. RNA was reversed transcribed using the SuperScript™ VILO™ Master Mix (Thermofisher #11755250). The cDNA was then subjected to 2-step thermocycling using Power up qPCR SYBR Green mix (Thermofisher # A25742) and data collection was performed on a Mic qPCR machine (Bio Molecular Systems). Expression levels were normalized using the ∆∆Ct method with *Actb* as the reference gene. The primers used were *Trpv1* Forward: GGCCGAGTTTCAGGGAGAAA; *Trpv1* Reverse: TATCTCGAGTGCTTGCGTCC; *Calca* Forward: AGGCACCGCTCACCAG; *Calca* Reverse: CCTGGGCTGCTTTCCAAGAT; *Actb* Forward: TGTCGAGTCGCGTCCACC; *Actb* Reverse: TATCGTCATCCATGGCGAACTGG; *Trpa1* Forward: TCCAAATTTTCCAACAGAAAAGGA; *Trpa1* Reverse: CGCTATTGCTCCACATTGCC; Trpm8 Forward: AGCAGTGGAGTTGTTCACCG, Trpm8 Reverse: GCTTCGCAGGAGTAGACCAG.

### Statistical analysis

Results are expressed as mean ± standard error of the mean (S.E.M.) in all experiments. The statistical significance was tested by one-way or two-way ANOVA, two-tail unpaired Student T-test, or Mann-Whitney test as indicated in figure legends. Values were considered significantly different when p < 0.05. Statistical computations and graphs were made with GraphPad 8.1 software (GraphPad Software).

## Supplementary Information

**Additional file 1: Supplementary Figure 1.** NGF does not impact gene transcription in cultured sensory neurons. Nociceptor neurons were harvested and cultured (5000 neurons/well) for 24h in the presence of Nerve Growth Factor (NGF, 50 ng/mL; denote*d as +*) or its vehicle (*denoted as -*). ∆∆Ct were calculated for *Trpv1* (**A**) and *Calca* (**B**) and fold change were calculated in comparison to untreated JNC neurons. NGF did not impact *Trpv1* (**A**) and *Calca* (**B**) gene expression in JNC or DRG neurons (**A, B**). *Data are shown as mean ± S.E.M. Number of dishes tested is shown. N=3 biological replicates/group. P-values were determined by unpaired Student's t-test.*

## Data Availability

All data generated during this study are included in this published article and its supplementary information files. Some of the data analyzed from previous publications were obtained from public database NCBI Gene Expression Omnibus (GSE141395 and GSE145216).

## Abbreviations

BDNF: Brain-derived neurotrophic factor
CGRP: Calcitonin gene-related peptide
DRG: Dorsal root ganglia
JNC: Jugular nodose ganglia
NGF: Nerve growth factor
SP: Substance P
Tac1: Tachykinin peptide 1
T_h_1: Type 1 helper cells
T_h_2: Type 2 helper cells
TRPA1: Transient receptor potential ankyrin subtype 1 protein
TRPM8: Transient receptor potential cation channel subfamily melastatin member 8
TRPV1: Transient receptor potential vanilloid type 1
VIP: Vasoactive Intestinal Peptide
