# Supplementary figures and images for: Anatomical differences in nociceptor neurons sensitivity
Source: Bioelectron Med. 2022 Apr 6;8:7. doi: 10.1186/s42234-022-00088-w (PMC8985299; doi:10.1186/s42234-022-00088-w)

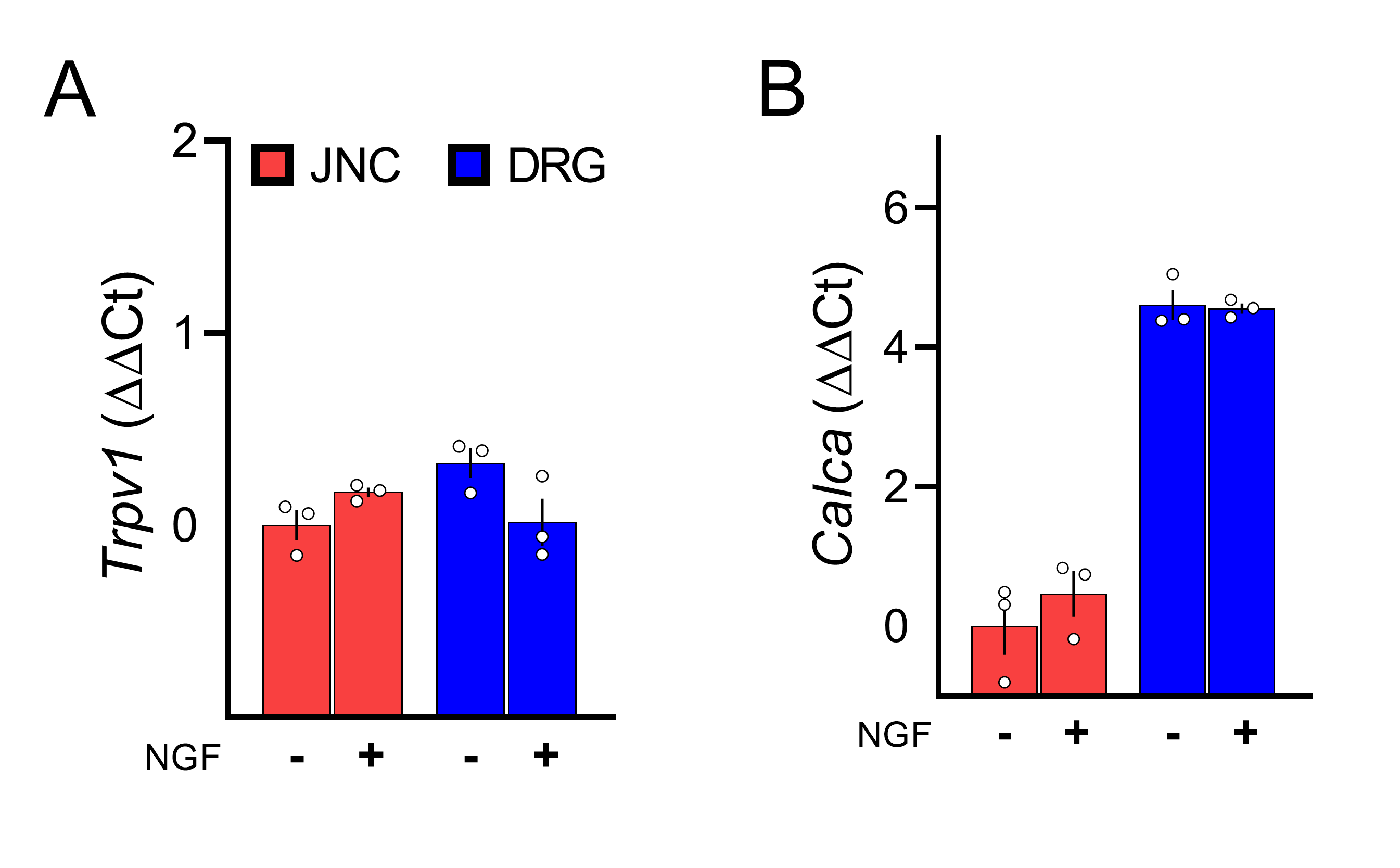

Supplement: Supplementary file 1 — Additional file 1: Supplementary Figure 1. NGF does not impact gene transcription in cultured sensory neurons. Nociceptor neurons were harvested and cultured (5000 neurons/well) for 24h in the presence of Nerve Growth Factor (NGF, 50 ng/mL; denoted as +) or its vehicle (denoted as -). ∆∆Ct were calculated for Trpv1 (A) and Calca (B) and fold change were calculated in comparison to untreated JNC neurons. NGF did not impact Trpv1 (A) and Calca (B) gene expression in JNC or DRG neurons (A, B). Data are shown as mean ± S.E.M. Number of dishes tested is shown. N=3 biological replicates/group. P-values were determined by unpaired Student's t-test. [file 42234_2022_88_MOESM1_ESM.tif]
